# Early intervertebral degeneration patterns among elite amateur golfers: A T2∗ mapping study

**DOI:** 10.1016/j.bas.2026.106154

**Published:** 2026-06-30

**Authors:** Tobias Mann, Daniel Benjamin Abrar, Bernd Bittersohl, Max Prost

**Affiliations:** a Department for Orthopedic and Trauma Surgery, University of Düsseldorf, Medical Faculty, Düsseldorf, Germany; b Department for Diagnostic and Interventional Radiology, University of Düsseldorf, Medical Faculty, Düssledorf, Germany; c Department for Orthopedics and Trauma Surgery, University of Bielefeld, Medical Faculty, Bielefeld, Germany

**Keywords:** Golf, intervertebral disc, Lumbar spine, MRI, T2∗ mapping, Disc degeneration, Pfirrmann grading, Sports biomechanics, Nucleus pulposus, Quantitative MRI, Low back pain, Proteoglycan

## Abstract

This study examined whether repetitive loading forces and overuse expose elite golfers to a heightened risk of early degenerative intervertebral disc (IVD) injuries. We hypothesized that asymptomatic elite golfers would demonstrate characteristic patterns of IVD degeneration on T2∗ magnetic resonance imaging (MRI) compared with asymptomatic non-golfers.

This pilot study enrolled ten high-level amateur golfers (mean age 28.2 ± 7.3 years) and ten asymptomatic non-golfers (mean age 28.6 ± 4.8 years). The control group was matched for BMI and had no history of competitive sport. MRI scans including T2∗ mapping were performed on a 3-T scanner. T2∗ values were compared between both groups across four lumbar IVDs; midsagittal measurements were obtained in five zones: anterior annulus fibrosus (AF), anterior nucleus pulposus (NP), central NP, posterior NP, and posterior AF. Pfirrmann grading was used for morphological analysis. Statistical analysis employed the Mann–Whitney *U* test, Wilcoxon matched-pairs test, and Spearman rank correlation coefficient. A Monte Carlo-based power analysis was performed to estimate the sample size required for adequately powered future studies.

No statistically significant difference in overall T2∗ values were found between groups (golfers: 48.61 ± 44.49 ms vs. controls: 45.67 ± 38.11 ms; P = 0.16). However, intersegmental and interzonal analyses revealed distinctive patterns of IVD degeneration in the golfer group. Golfers also demonstrated a higher frequency of elevated Pfirrmann grades (1-4) compared with controls. Monte Carlo simulations indicated that approximately 140-180 participants per group would be required to achieve 80% statistical power for the L5/S1 segment, while several regions, including the anterior and posterior annulus fibrosus and posterior nucleus pulposus, would require more than 1000 participants per group.

Although overall T2∗ values did not differ significantly between cohorts, the observed tendencies suggest early signs of disc degeneration potentially attributable to the high biomechanical demands of golf. These pilot findings lay the groundwork for larger, adequately powered investigations.

**Study design:**

Cross-sectional study; Level of evidence, 3.

## Highlights

• T2∗ mapping explored early lumbar disc changes in elite golfers.
• No statistically significant overall T2∗ differences vs matched non-golfers.
• Segmental and zonal differences suggest early degeneration patterns.
• Findings support biomechanical stress as a factor in disc health.

## 1 Introduction

Golf, a sport practiced by around 55 million people worldwide, offers numerous mental and physical health benefits (Murray et al., 2017; Farrally et al., 2003). Known for fostering concentration, patience, and strategic thinking, golf also provides cardiovascular exercise, particularly through walking, and a form of moderate physical activity that promotes musculoskeletal health. However, despite its reputation as a low-impact sport, golf involves complex biomechanics that can lead to specific injury patterns, particularly among skilled and professional players. Recent studies have highlighted the need to examine these patterns, especially regarding the impact of repetitive spinal movements and high swing speeds on the lumbar spine, as many injuries in golf are linked to overuse rather than acute incidents (McHardy et al., 2007a; Gosheger et al., 2003; Burdorf et al., 1996; Zouzias et al., 2018) (see Fig. 9, Fig. 10, Fig. 11, Fig. 12)

Epidemiological data on golf injuries is still limited, with only a few studies focusing on injury prevalence and risk factors. One such study by Mc Hardy et al. found that 15.8 injuries occur per 100 golfers annually, equating to 0.36-0.6 injuries per 1000 h per person (McHardy et al., 2007a). While these numbers suggest a relatively low risk for recreational golfers, further research has indicated that professionals, with higher training loads and repetitive stress, experience injury rates that are significantly higher than those of amateurs. A study by Gosheger et al. noted that 82.6% of golf injuries in professionals were attributed to overuse, primarily affecting the back and lumbar regions (Gosheger et al., 2003). The golf swing, requiring considerable strength and torque, places substantial stress on the spine, particularly in the lumbar area, where repetitive twisting, bending, and compressive forces are applied (Zouzias et al., 2018). To standardize the assessment of golf-related injuries and guide future research, an international consensus statement on injury and illness surveillance methodology in golf has been established, defining standardized definitions, data collection procedures, and reporting templates for prospective epidemiological studies (Murray et al., 2020). This framework has since been applied in large-scale cohorts: a cross-sectional survey of 1170 male golfers reported the prevalence and characteristics of musculoskeletal complaints across playing levels (Murray et al., 2023), and a prospective season-long study of 910 amateur golfers further characterized injury incidence and patterns using this consensus methodology (Robinson et al., 2024). Together, these studies underscore the high burden of musculoskeletal complaints in golfers across competitive levels, and provide a methodological foundation upon which imaging-based pilot studies such as ours can be contextualized.

The mechanics of the golf swing are complex, with critical elements such as the "X-Factor" and the "Crunch Factor" playing a significant role in both performance and injury risk (Bourgain et al., 2022). The X-Factor measures the separation between the shoulders and hips during the backswing, generating power that can increase clubhead speed (Gluck et al., 2008). Meanwhile, the Crunch Factor describes the compressive force exerted on the spine at impact and early follow through. These biomechanical factors help golfers achieve high speeds and distances, but they also elevate the risk of injury due to the increased forces on the lumbar spine. For professional golfers, these demands are amplified as they regularly achieve clubhead speeds upwards of 115 mph, with PGA Tour averages reaching 115.94 mph in 2024, compared to an average of 93.4 mph for amateurs. (PGA Tour) This heightened workload contributes to a higher prevalence of injury among professional golfers (Chu et al., 2010).

One of the most concerning types of injury for golfers is lumbar intervertebral disc (IVD) degeneration. The intense forces exerted on the spine during the golf swing can lead to wear on these discs, particularly under conditions of high compression, shear, lateral bending, and rotational loads. These forces can reach up to eight times a golfer's body weight, posing significant risks for lumbar disc health (Hosea and Gatt, 1996a; McHardy et al., 2006). Among the biomechanical factors contributing to these forces, the X-Factor and Crunch Factor are commonly examined. Although these mechanics are beneficial in terms of performance, they are also linked to a heightened risk of low-back pain and lumbar spine injuries. According to research by Gluck et al., the repetitive nature of these forces, combined with extreme spine mechanics, increases the likelihood of IVD injuries, especially among professional and high-level amateur golfers who train intensively (Gluck et al., 2008).

This study aims to deepen the understanding of lumbar spine health in golfers by investigating the intervertebral discs of high-level players using advanced imaging techniques. We hypothesized that the repetitive spinal torsion, axial loading, and vertical compression inherent in the golf swing increase the risk of early degenerative changes in the lumbar intervertebral discs for high-level golfers. To test this hypothesis, a pilot study was conducted comparing magnetic resonance imaging (MRI) and T2∗ relaxometry data of high-level amateur golfers with that of a control group of asymptomatic non-golfers. T2∗ mapping, a biochemical MRI technique sensitive to early degenerative changes, offers new insights into the condition of the lumbar discs, potentially revealing microstructural changes not visible on conventional MRI alone (Hesper et al., 2014).

T2∗ mapping offers several specific advantages that make it particularly suited to early detection of disc degeneration. First, it is quantitative and continuous, providing a numerical relaxation value per voxel or region of interest rather than a categorical morphological grade, allowing detection of subtle, graded biochemical change rather than only binary presence/absence of degeneration. Second, T2∗ values correlate closely with proteoglycan and water content within the nucleus pulposus and with collagen fiber organization in the annulus fibrosus, both of which decline early in the degenerative cascade, often years before disc height loss, signal change, or herniation become visible on T1-or T2-weighted sequences (Roberts et al., 2006). Third, the technique requires no contrast agent, unlike dGEMRIC, making it safe for repeated use in healthy, asymptomatic athletes and suitable for longitudinal monitoring across a training season or career (Bittersohl et al., 2013). Fourth, T2∗ mapping has a relatively short acquisition time (in our protocol, approximately 11 min for a high-resolution three-dimensional dataset), making it feasible to incorporate into research and potentially clinical screening protocols for athletes without excessive scan-time burden. Beyond the intervertebral disc, T2∗ mapping and related multi-echo gradient-echo techniques have demonstrated broad utility across musculoskeletal and neurological imaging. In articular cartilage, T2∗ mapping is sensitive to collagen network integrity and has been used to characterize early osteoarthritic change in the hip, knee and shoulder, often before changes are detectable with conventional morphological MRI (Mosher et al., 2000). In the spine, T2∗-weighted gradient-echo sequences are useful for detecting facet joint cartilage degeneration, which frequently coexists with disc degeneration and contributes to overall segmental instability (Bittersohl et al., 2012). In the spinal cord, susceptibility-sensitive T2∗/gradient-echo sequences are valuable for identifying microhemorrhages following trauma and for characterizing hemosiderin deposits in conditions such as cavernous malformations (Haacke et al., 2009). In neurodegenerative disease, T2∗ and related susceptibility-weighted techniques are used to quantify iron deposition in deep gray matter structures, which has been proposed as an imaging biomarker of disease progression in conditions such as Parkinson's disease (Haacke et al., 2009). This versatility, spanning cartilage, disc, cord, and brain, reflects the common underlying sensitivity of T2∗-based techniques to tissue water content, macromolecular organization, and paramagnetic substances, and supports its use as a generalizable quantitative biomarker across musculoskeletal and neuroradiological applications.

The findings from this study could have broad implications for golf training and injury prevention. By identifying early markers of lumbar degeneration in golfers, especially those who train at high intensities, it may be possible to develop targeted strategies for preserving spinal health. Such strategies could include optimized warm-up routines, biomechanical training focused on minimizing spinal stress, and personalized fitness programs that strengthen the spine without excessive loading. For both amateur and professional golfers, these approaches could improve performance and reduce injury risk, promoting longevity in the sport. Ultimately, this research not only aims to better understand the biomechanics of golf-related spinal injuries but also to contribute to a body of knowledge that will help both medical professionals and athletes make informed decisions about training and injury prevention in golf.

## 2 Methods

This study employed a two-step design. First, it adapted the imaging and analysis protocol previously validated by Benedikter et al. (2022) to pilot the assessment of disc degeneration in golfers using a small sample. Second, the resulting pilot data were used to perform a Monte Carlo simulation to estimate the sample size required for an adequately powered future study.

### 2.1 Study population

The study, which received approval from the local ethics committee, involved ten partly symptomatic high-level amateur golfers (0 women, 10 men; 10 right-handed, 0 left-handed; mean age, 28.20 ± 7.28 years; age range, 22 - 42 years). These individuals, who met the criteria of a high-level amateur golfer, including a handicap of 2 or better and a minimum of 5 years of competitive Golf with an average Training commitment of at least 10 h/week, participated voluntarily. The average clubhead speed with the Driver was 114.4 ± 5.52 mp/h, ranging from 100 to 124 mp/h. The golfers were recruited from different Teams in Germany, primarily from North-Rhine Westfalia. All Teams are participating in the DGL (Deutsche Golf Liga) and play in the Regional-, 1.- and 2. Bundesliga. The mean body mass index (BMI) was 23.98 ± 1.52 kg/m2 (range, 21.30 – 26.00 kg/m2). We excluded all golfers who did not meet our requirements concerning high-level amateur golf and golfers with contraindications to MRI or any history of spinal surgery.

The control group included ten asymptomatic non-golfers (0 women, 10 men; mean age, 28.60 ± 4.84 years; age range, 25 - 39 years) who had never practiced sport on a competitive level, especially rotational sports like tennis or golf. They did not exercise more than 5 h a week in any sport and had no history of spine surgery or spine complaints. The control group was chosen to provide a baseline for comparison with the study population, as they represent individuals who do not engage in the high-impact, repetitive movements characteristic of golf. The mean BMI was 22.64 ± 1.89 kg/m2 (range, 20.50 – 26.60 kg/m2). The control group was recruited from the area and designed to match the golfers’ BMI. The non-golfers took part voluntarily as well. Before undergoing an MRI, all participants underwent a comprehensive physical examination. This physical examination included an investigation of pain, discomfort, tenderness, and range of movement.

**Fig. 1:**
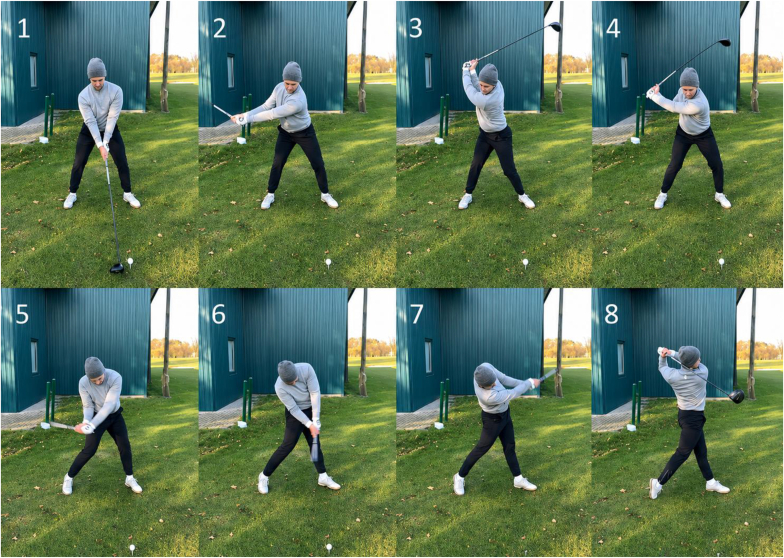
Golfswing Sequence of an elite golfer. (1) The address position features flexed knees and hips, with weight evenly distributed between both feet, which are pointed outward to increase hip rotation range. The spine is slightly flexed, with a mild lateral bend in the thoracic spine away from the target. These angles in the spine, hips, and knees must be maintained throughout the swing, as they directly affect the swing plane. (2) The golf swing begins with rotating the hips and torso away from the target. Hip rotation is driven by ground shear forces produced by the feet: pushing the right foot forward and the left foot backward creates counter-clockwise shearforce in the backswing. For the downswing, this is reversed. These shearforces produce opposite torque, leading to pelvis rotation. Weight is loaded onto the right side, while the lumbar spine laterally flexes and extends toward the target. The arms remain connected and wide in front of the body. (3) At the top of the backswing, the player has rotated the hips about 45° and the spine an additional 50°, totaling 95 degrees of upper body rotation. The difference between pelvis and torso rotation, or the X-Factor, enhances power. The lumbar spine extends and bends toward the target. The player lifts the arms in relation to his chest and momentarily shifts weight to a 70:30 ratio on the right foot. (4) The early downswing starts by shifting weight to the left foot and rotating the hips toward the target. The torso follows with a slight delay, increasing the X-Factor and putting rotational stress on the spine. To keep the upper body centered, the lumbar spine flexes laterally away from the target and reduces its extension. Modern golf swings use ground forces to boost clubhead speed, illustrated by the player pushing weight into the ground by increasing hip and knee flexion. (5) In the late downswing, the player rotates the hips toward the target while transferring weight to the left foot. The torso follows the hip rotation, maintaining a constant X-Factor, continuing to apply rotational stress to the spine. Just before impact, the player begins to push off the ground, creating vertical pressure on the spine, particularly on the posterior part of the IVD due to the physiological lumbar lordosis. The lumbar spine's lateral flexion away from the target increases pressure on the right side of the IVD. To maintain posture angles and avoid shifting the swing plane, the player must increase lateral bend toward the impact position. This lateral bend, however, increases the crunch factor, which is defined as the product of the torsos' lateral inclination angle and the torsos' axial rotation speed concerning the pelvis. It was first described by Sugaya et al. (Sugaya et al., 1996) (6) At impact, the hips are rotated about 70° toward the target, while the delayed torso rotation still creates the X-Factor. The player continues to push off the ground by extending the hips and knees, causing the left foot to lift off the ground. The lumbar spine increases lateral flexion, while vertical forces on the IVD increase. The thoracic and cervical spine begin to crunch due to arm extension and centrifugal forces. (7) Post impact, the player continuously turns through the ball while the torso rotation catches up with the hip rotation. Due to centrifugal forces, both arms are fully extended. The lumbar spine starts extending and decreasing the lateral bend. (8) At finish, the weight should be on the left foot, and the torso and hips should be rotated at least towards the target line. The release of almost all lateral bends in the lumbar spine signifies a strong and stable finish. Before reaching the finish position, the player must stop the golf club's momentum, which produces rotational forces and causes stress to the IVD.

Golf swing sequence of a high-level amateur (Fig. 1).

### 2.2 Back pain and Oswestry Disability Index questionnaire

Golfers and non-golfers undergoing the MRI examinations completed a questionnaire assessing functional impairments, following Fairbank et al. (1980), before the examination. The Oswestry Disability Questionnaire (ODI) offers a subjective performance score in daily activities, consisting of 10 questions, each graded from 0 to 5 (5 = the highest disability level). The total scores are expressed as percentages (0%-20% reflecting minimal disability, 81%-100% indicating severe disability). This questionnaire, employed in various studies involving athletes, has demonstrated its utility (Benedikter et al., 2022; Baranto et al., 2009; Sward et al., 1991; Witwit et al., 2018). Additionally, the golfers answered a specific questionnaire that offered additional details on their golf-related skills and training habits.

### 2.3 Magnetic resonance imaging

All participants had MRI scans conducted in the afternoon between 4 p.m. and 6 p.m. All participants in the study underwent MRI while lying on a 3-T scanner (Prisma; Siemens Medical Solutions) in a supine position. We integrated a spinal matrix coil (24-channel, triple mode) into the patient table. The MRI protocol included standard sequences (localizer images, T1-and T2-weighted transverse and sagittal MRI scans), each with a 4 mm slice thickness. A high-resolution three-dimensional multi-echo data image combination (MEDIC) sequence was included. The MEDIC sequence featured the following imaging parameters: repetition time = 43 ms; echo time = 5, 10, 15, 20, 25, and 30 ms; field of view = 192 × 216 mm2; slice thickness = 1 mm; voxel size = 1 × 1 × 1 mm3; slice gap = 1.2 mm; receiver bandwidth = 260 Hz per pixel; flip angle = 25°; number of excitations = 1; and scan time = 11 min 10 s. The T2∗ maps underwent automatic in-line processing (SyngoMapIT; Siemens Medical Solutions) utilizing a nonlinear, squared, curve-fitting algorithm (Benedikter et al., 2022).

### 2.4 Postprocessing, morphological analysis and T2∗ assessment

**Fig. 2:**
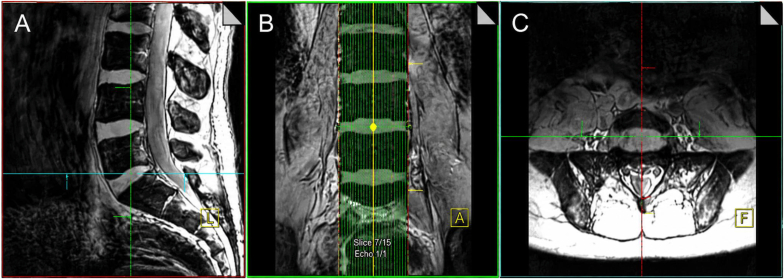
Multiplanar reconstruction of the T2∗-weighted data mapping set, illustrating the (A) sagittal, (B) coronal and (C) transversal planes.

Image processing and T2∗ assessments were conducted using a Leonardo working station. Therefore, midsagittal planes with a slice thickness of 4 mm were generated through multiplanar reformatting (Fig. 2). Four disks of the lumbar spine (segments L2-L3, L3-L4, L4-L5, and L5-S1) were examined as the IVD between the first and second lumbar vertebrae was excluded because T2∗ assessment in the thoracolumbar region is affected by motion artifacts due to respiratory motion and vascular pulsations from the heart and aorta (Benedikter et al., 2022). A radiologist (D.B.A.) with seven years of practice performed the morphological assessment, assigning Pfirrmann grades to each IVD based on the morphological T2∗-weighted images. T2∗ assessment was conducted by means of region of interest (ROI) analysis by a biochemical MRI expert with 15 years of experience.

**Fig. 3:**
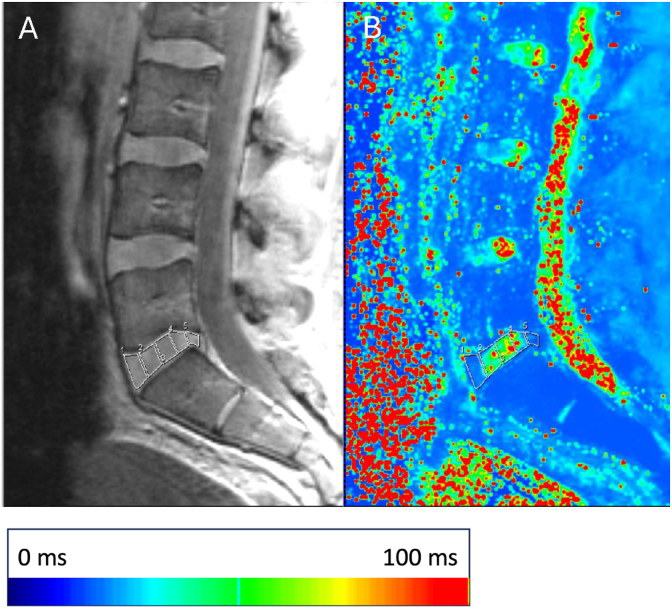
(A) Midsagittal reformat of T2∗-weighted morphological magnetic resonance imaging and (B) the corresponding midsagittal T2∗ map. Five regions of interest were identified in the sagittal direction: anterior annulus fibrosus (AF), anterior nucleus pulposus (NP), central NP, posterior NP and posterior AF. Note: The observer tried to create 5 zones manually, that reflected the anatomy of the IVD and were roughly proportioned in the sagittal plane. However due to the manual creation, the ROIs were not equal in size, neither between nor within a single IVD level. In addition, the disk shape is not rectangular and exhibits variations in height in the sagittal course. To ensure a reliable measurement of tissue within the IVD, the ROIs did not strictly adhere to the exact contours of the IVD. As a result, a few pixels of potential IVD tissue may have been excluded from the ROIs.

**Fig. 4:**
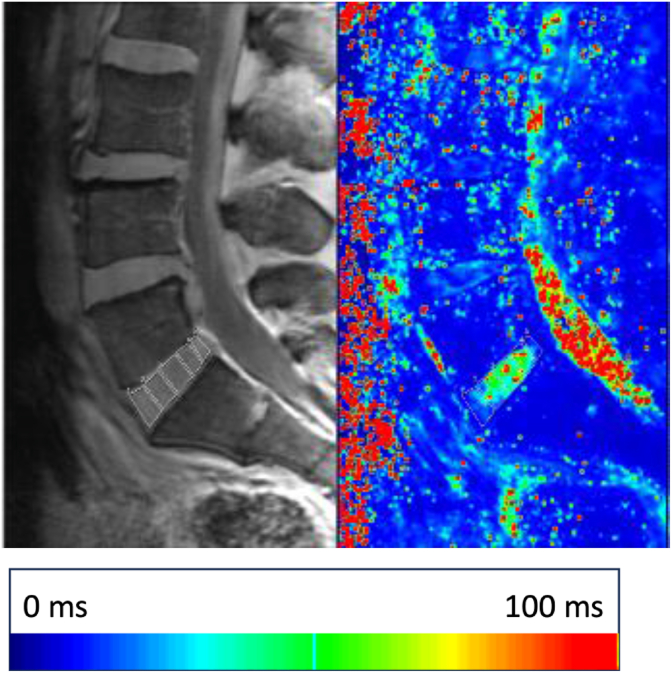
(Left) Midsagittal reformat of T2∗-weighted morphological magnetic resonance imaging and (right) the corresponding midsagittal T2∗ map. Five regions of interest were identified in the sagittal direction: anterior annulus fibrosus (AF), anterior nucleus pulposus (NP), central NP, posterior NP and posterior AF. Note: Low T2∗ values can be observed in L2/3 - L4/5 as signs of early degeneration.

T2∗ values were acquired from 5 ROIs, manually drawn on both the sagittal morphological T2∗-weighted images and the corresponding T2∗ maps, effectively dividing the IVD into five zones: the anterior annulus fibrosus (AF), anterior nucleus pulposus (NP), central NP, posterior NP, and posterior AF. The ROIs on the morphological T2∗-weighted images guided the placement of ROIs on the T2∗ maps. The T2∗-weighted images and their corresponding T2∗ maps were presented in a dual-screen layout image area, ensuring optimal visibility of details with appropriate contrast and brightness. Using a freehand drawing tool, the ROIs were initially outlined on the T2∗-weighted image. The drawing was automatically transferred to the T2∗ map through a copy-and-paste approach, where both images were selected simultaneously. Subsequently, the ROI outlines were reassessed in the T2∗ map and adjusted minimally if necessary to correct for any offset. The reliability of this method, as previously reported, demonstrated substantial agreement in T2∗ measurement (Bittersohl et al., 2012, 2013, 2019; Kolf et al., 2019). A single person performed the T2∗ measurement (Fig. 3) (see Fig. 4).

### 2.5 Statistical analysis

The statistical analysis for this study was performed by a biostatistician who used already proven methods like in Benedikter et al. (2022). The study and control groups were compared regarding sex, age, and BMI using the Student's t-test to identify any significant differences. Intra- and interrater reliability for Pfirrmann grading was evaluated for consistent agreement using Spearman's rank correlation (r), where a strong relationship is indicated when r > 0.7, moderate when 0.5 < r < 0.7, mild when 0.3 < r < 0.5, and very weak when r < 0.3. Mean T2∗ values with standard deviation and the 95% confidence interval (CI) were reported. The comparison of T2∗ values between the study and control groups employed the Mann-Whitney *U* test. These T2∗ comparisons were performed globally, by segment, and by zone. The correlation between T2∗ values and Pfirrmann grades was determined using Spearman's rank correlation. Data was initially entered into an Excel spreadsheet (Version 14, Microsoft Office Professional; Microsoft) and transferred to SPSS software (Version 25; IBM). A significance level of P < 0.05 was considered statistically significant. Summary statistics (means and standard errors) obtained from the pilot dataset (n = 10 per group) were used to derive group-specific standard deviations.

This simulation-based approach to power estimation follows established methodology for situations in which closed-form power formulae are unavailable or impractical for the specific distributional assumptions and test statistics employed (Arnold et al., 2011). For each region, datasets were simulated under both normal and lognormal distributional assumptions. Sample sizes ranging from 10 to 1000 participants per group (increments of 10) were evaluated. For each sample size, 1000 Monte Carlo simulations were generated for the golfer and control groups using the observed means and estimated standard deviations. Group differences were tested using the Wilcoxon rank-sum test with a two-sided significance level of α = 0.05. Statistical power was calculated as the proportion of simulated datasets yielding a significant result (p < 0.05). The minimum sample size required to achieve 80% and 90% power was determined for each anatomical region under both distributional assumptions. All simulations were performed in R (version 4.5.1).

## 3 Results

In total, 80 IVDs (10 control, 10 golfers, four disks from L2 to S1, and 400 IVD zones) were included in the study. Due to artifacts, 1 zone must be excluded in the golfers T2∗ data set. In the control group, 3 IVDs with 15 zones needed to be excluded from the T2∗ data set because of artifacts. Therefore, 40 golfers‘ IVDs with 199 zones were compared to 37 control IVDs, including 175 zones.

Both the golfer and control groups were similar regarding sex, age, and BMI (Table 1). The physical examination concerning Schober and Ott's measurements also showed no statistical differences. All volunteers answered the Oswestry Disability Questionnaire. The control group's mean score was 0.4%, compared to the golfer group's score of 5%. No further statistical analysis was conducted.

**Table 1:** Characteristics of the study groups.

	Controls (n = 10)	Golfers (n = 10)	*P*
Female	0	0	
Male	10 (100.0)	10 (100.0)	
Age,y	28.60 ± 4.84 (25-39)	28.20 ± 7.28 (22-42)	0.89
BMI, kg/m2	22.64 ± 1.89 (20.50-26.60)	23.98 ± 1.52 (21.30-26.00)	0.10

Note: Data are presented as n (%) or mean ± SD (range). BMI, body mass index. Bolding indicates a statistically significant difference between groups (P < 0.05).

The Inter-Observer Reliability was established with an absolute intraclass correlation from 0.97 to 0.98.

The mean ROI (cm2) ranged from 0.245 to 0.756 in the control group and 0.223 to 0.725 in the golfer group. The mean overall T2∗ values showed no statistical difference between both groups (Golfer: 48.61 ± 44.49 ms vs Control: 45.67 ± 38.11 ms; *P* = 0.16).

The intersegmental comparison showed higher mean T2∗ values for the golfer groups IVDs from L2/L3 to L4/L5. Only the IVD L5/S1 showed lower mean T2∗ values than the control group. The pairwise comparison exposed no statistically significant differences for all segments (Table 2).

**Table 2:** Intersegmental comparison of mean T2∗ Times (in ms) between the study groups.

Region	Group	Mean	Std. Error	df	95% Confidence Interval		*P*
					Lower Bound	Upper Bound	
L2-3	Golf	44.92	4.51	40.37	35.81	54.02	0.06
	Control	32.04	4.75	40.21	22.44	41.63	
L3-4	Golf	44.06	4.32	40.18	35.34	52.78	0.75
	Control	42.07	4.55	40.18	32.88	51.27	
L4-5	Golf	55.06	5.70	49.69	43.60	66.51	0.86
	Control	53.56	6.01	49.69	41.48	65.63	
L5-S1	Golf	49.74	4.36	52.94	40.99	58.50	0.49
	Control	54.08	4.36	52.94	45.32	62.83	

Note: Bolding indicates a statistically significant difference between groups (P < 0.05). IVD, intervertebral disk; ms, milliseconds.

**Fig. 5:**
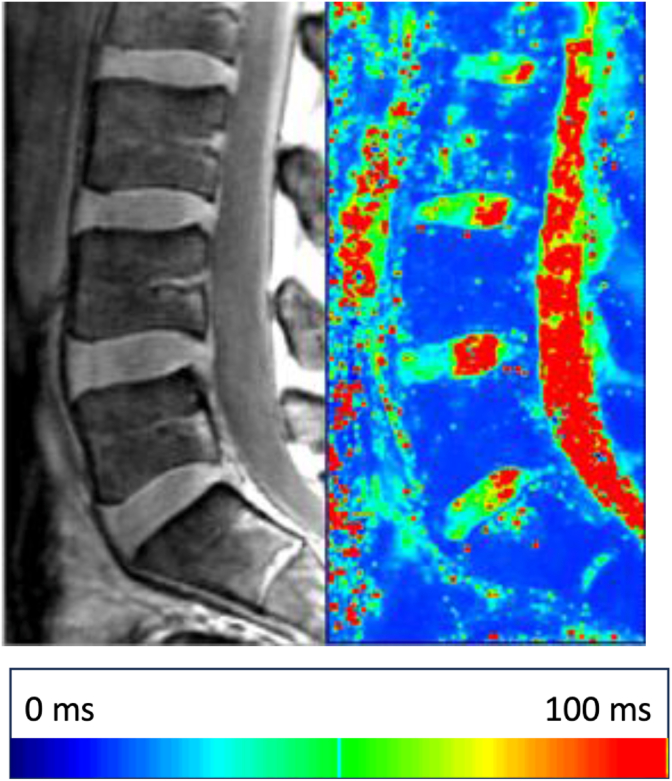
(Left) Midsagittal reformat of T2∗-weighted morphological magnetic resonance imaging and (right) the corresponding midsagittal T2∗ map. Note: High T2∗ values can be observed among L2/3 - L5/S1 as signs of healthy IVDs.

The interzonal comparison showed higher mean T2∗ values for the elite golfers in the nucleus pulposus anterior and the nucleus pulposus central compared to the control group. The golfer's group's lower mean T2∗ values were detected in the nucleus pulposus posterior. Only marginal differences were found in the anterior and posterior annulus fibrosus. The conducted pairwise comparison showed all differences were not statistically significant (Table 3) (see Fig. 5).

**Table 3:** Interzonal comparison of mean T2∗ Times (in ms) between the study groups.

Zone	Gruppe	Mean	Std. Error	df	95% Confidence Interval		*P*
					Lower Bound	Upper Bound	
annulus fibrosus anterior	Golf	20.85	1.53	63.00	17.79	23.91	0.66
	Control	21.81	1.57	62.59	18.66	24.96	
nucleus pulposus anterior	Golf	62.77	5.22	57.71	52.32	73.22	0.15
	Control	51.88	5.43	57.08	41.00	62.76	
nucleus pulposus central	Golf	81.38	7.45	59.99	66.48	96.28	0.41
	Control	72.50	7.76	59.26	56.97	88.02	
nucleus pulposus posterior	Golf	58.73	7.43	60.34	43.87	73.59	0.66
	Control	63.44	7.77	59.40	47.91	78.98	
annulus fibrosus posterior	Golf	18.49	1.08	59.53	16.33	20.66	0.55
	Control	17.55	1.13	58.65	15.28	19.81	

Note: Bolding indicates a statistically significant difference between groups (P < 0.05). IVD, intervertebral disk; ms, milliseconds.

**Fig. 6:**
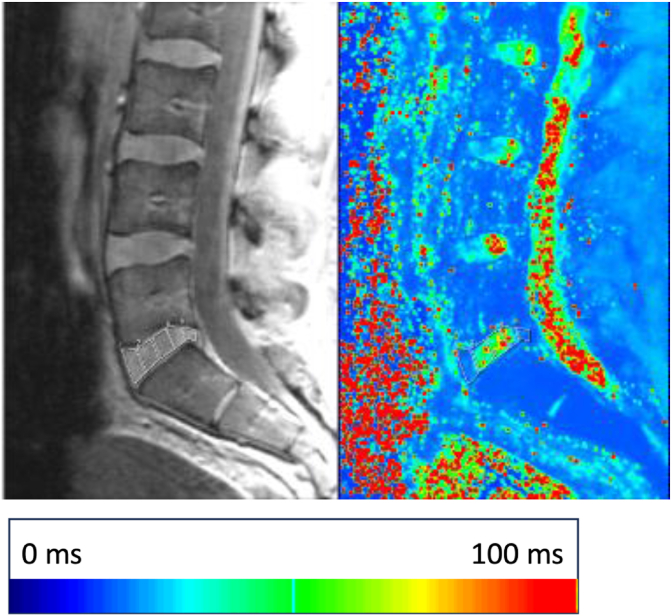
(Left) Midsagittal reformat of T2∗-weighted morphological magnetic resonance imaging and (right) the corresponding midsagittal T2∗ map. Five regions of interest were identified in the sagittal direction: anterior annulus fibrosus (AF), anterior nucleus pulposus (NP), central NP, posterior NP and posterior AF. Note: High T2∗ values can be observed among L2/3 - L5/S1 as signs of healthy IVDs.

We compared the Pfirrmann grades in the elite golfers' IVDs to those of our control group (Table 4). The control groups‘IVDs were classified from Pfirrmann 1 to 3, while the golfers showed grades from 1 to 4, with a higher frequency of high gradings (Fig. 6). The T2∗ values per Pfirrmann grades are illustrated in Fig. 6.

**Table 4:** Pfirrmann grade distribution in controls and elite golfers a.

Pfirrmann Grade b	Controls	Golfers
1	18 (45%)	8 (20%)
2	18 (45%)	24 (60%)
3	5 (12,5%)	6 (15%)
4	0 (0%)	2 (5%)
5	0 (0%)	0 (0%)
Total	40 (100%)	40 (100%)

Note.

a Data are presented as No. of intervertebral disks (IVDs) (%).

b Grade 1 = homogeneous, bright white, IVD height normal; grade 2 = inhomogeneous with or without horizontal bands, IVD height normal; grade 3 = inhomogeneous, gray, unclear distinction of nucleus and annulus, normal to slightly decreased IVD height; grade 4 = inhomogeneous, gray to black, no distinction of nucleus and annulus, IVD height normal to moderately decreased; grade 5 = inhomogeneous, black, no distinction of nucleus and annulus, IVD collapsed. (Pfirrmann et al., 2001).

Higher grades were revealed in the segments L4/5 and L5/S1 in the golfer's cohort. Both groups' differences demonstrated statistical significance in the segment L4/5 (2.20 ± 0.42 vs 1.60 ± 0.70; *P* = 0.03). All other segments couldn't display any statistically significant differences.

**Fig. 7:**
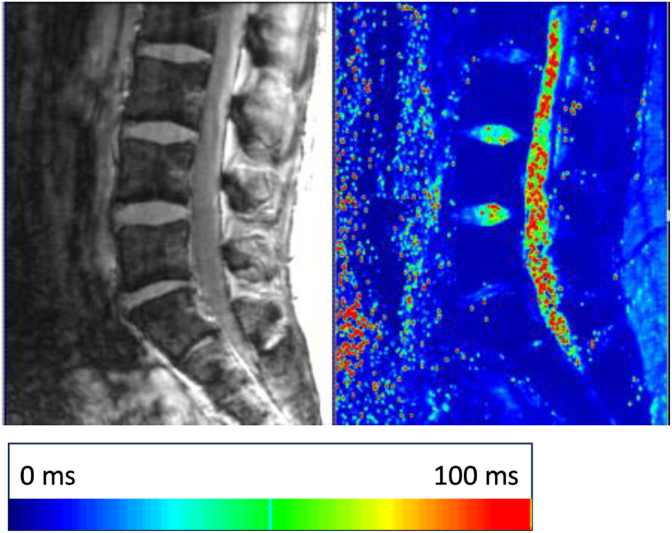
(Left) Midsagittal reformat of T2∗-weighted morphological magnetic resonance imaging and (right) the corresponding midsagittal T2∗ map. Note: Low T2∗ values are found in IVDs L2/3 and L5/S1 as signs of early degeneration. In comparison L3/4 and L4/5 show high values as signs of healthy IVDs.

**Fig. 8:**
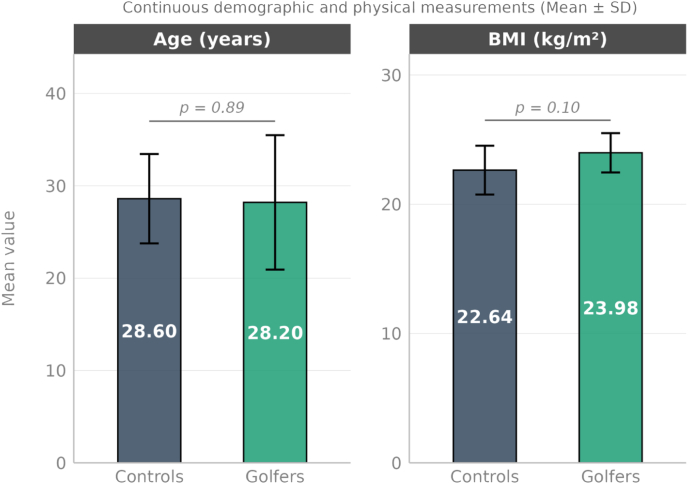
Characteristics of the study groups.

**Fig. 9:**
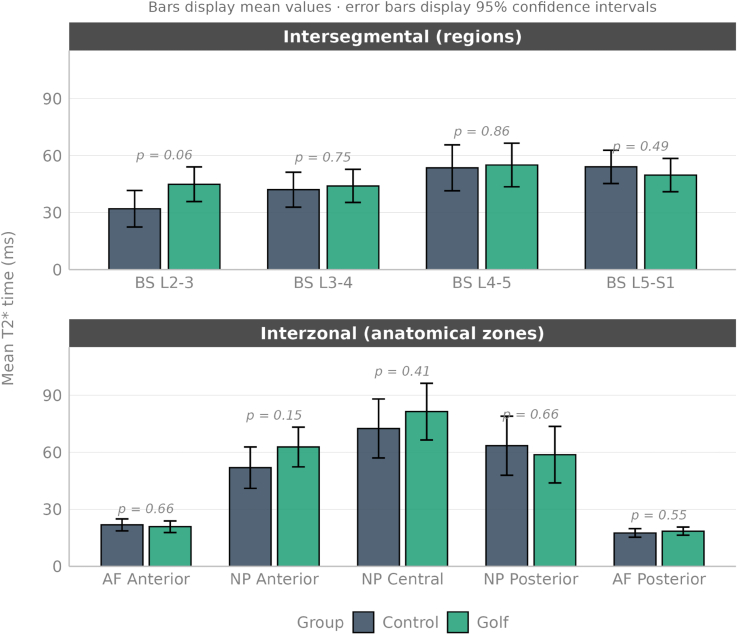
Comparison of Mean T2∗ Relaxation Times between study groups.

**Fig. 10:**
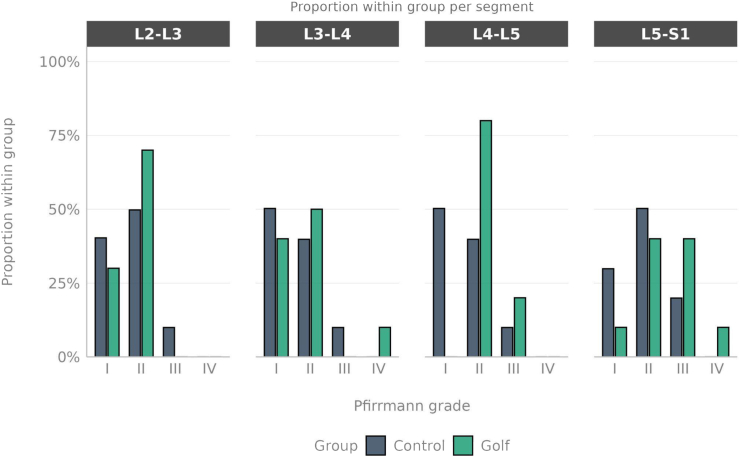
Distribution of Pfirrmann degeneration grades across lumbar disc levels in elite golfers and controls. Bars show the proportion of participants within each group assigned to each Pfirrmann grade (I–IV) at the L2–L3, L3–L4, L4–L5, and L5–S1 segments.

**Fig. 11:**
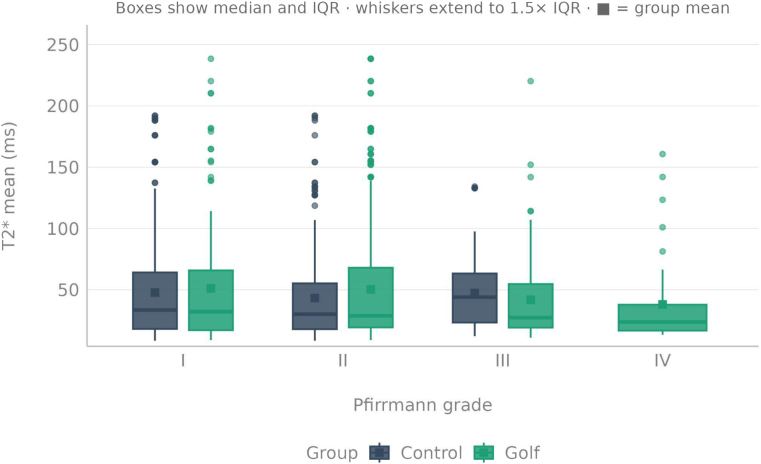
Mean T2∗ times by Pfirrmann grade in elite golfers and controls. Note: The boxes indicate the median and interquartile range, the whiskers indicate the minimum and maximum values. ms, milliseconds.

**Fig. 12:**
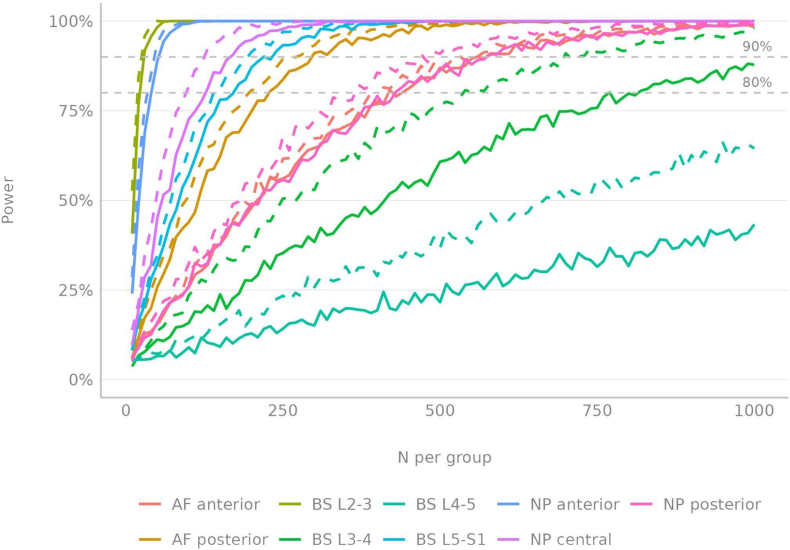
Note: Simulation-based Statistical Power Curves for Regional Group Comparisons. Statistical power curves across nine anatomical zones/regions, derived from 1000 independent Monte Carlo simulations per sample size configuration. Power (y-axis) is plotted as a function of sample size per group (x-axis, N = 10 to 150, evaluated in increments of 10) for a two-tailed Mann-Whitney *U* test (alpha = 0.05). Group means and standard deviations were parameterized using pilot study estimates (N orig = 10) comparing golf and control cohorts. Solid lines denote simulated data drawn from a symmetric normal distribution; dashed lines denote data drawn from a skewed log-normal distribution with equivalent arithmetic mean and variance. Horizontal reference lines indicate standard power thresholds at 80% and 90% power, defining the minimum required sample size (N) to reliably detect group differences within each unique anatomical region.

The simulation-based power analysis demonstrated substantial variability in the sample sizes required to detect between-group differences in T2∗ relaxation times across anatomical regions (Table 5, Fig. 7. The smallest sample sizes were required for the L2-3 region and the anterior nucleus pulposus, where 20-30 participants per group were sufficient to achieve 80% power and 30 participants per group were sufficient to achieve 90% power under both distributional assumptions. The central nucleus pulposus required 100-130 participants per group for 80% power and 130-170 for 90% power. For the L5-S1 segment, 140-180 participants per group were required to achieve 80% power and 200-220 for 90% power. The posterior annulus fibrosus required 200-230 participants per group for 80% power and 260-300 for 90% power. Several regions did not reach the predefined power thresholds within the simulated range of up to 1000 participants per group, including the anterior annulus fibrosus (390-420 for 80% power), posterior nucleus pulposus (370-430 for 80% power), L3-4 (540-770 for 80% power, with the normal distribution failing to reach 90% power within range), and L4-5, which did not achieve either threshold under any distributional assumption (see Fig. 8, Fig. 9, Fig. 10, Fig. 11, Fig. 12).

**Table 5:** Results of the power simulation.

Zone/Region	N for 80% power		N for 90% power	
	normal	lognormal	normal	lognormal
annulus fibrosus anterior	420	390	560	530
annulus fibrosus posterior	230	200	300	260
nucleus pulposus anterior	50	40	60	50
nucleus pulposus central	130	100	170	130
nucleus pulposus posterior	430	370	580	470
L2-3	30	20	30	30
L3-4	770	540	∗	700
L4-5	∗	∗	∗	∗
L5-S1	180	140	220	200

Note.

Statistical power was estimated using Monte Carlo simulation based on the observed group means and standard deviations for each anatomical region. Standard deviations were reconstructed from the reported standard errors. For each region, data were simulated under both normal and log-normal distributional assumptions using the observed summary statistics for the golfer and control groups. Sample sizes ranging from 10 to 1000 participants per group (in increments of 10) were evaluated. At each sample size, 1000 independent datasets were generated per group, and between-group differences were tested using a two-sided Wilcoxon rank-sum test at a significance level of α = 0.05. Statistical power was defined as the proportion of simulations yielding a statistically significant result. For log-normal simulations, the observed means and standard deviations were converted to the corresponding log-scale parameters prior to data generation. ∗ indicates that statistical power did not reach the predefined threshold of 80% or 90% within the simulated sample size range of 10 to 1000 participants per group.

## 4 Discussion

Our pilot study on the lumbar spine health of elite golfers using T2∗ mapping represents a novel approach to identify early signs of disc degeneration in a high-demand golf population. Golf, with its specific biomechanical demands, is often associated with both mental and physical health benefits, but it also presents unique risks for chronic injuries, particularly in the lumbar spine. Through this study, we sought to explore these risks and understand the subtle structural changes that may indicate early degeneration in golfers compared to a healthy control group. Although the results did not reach statistical significance due to the small sample size, the study reveals noteworthy trends that prompt further research and could potentially influence injury prevention and management strategies for golfers.

T2∗ mapping is an MRI technique that captures molecular mobility and water content in tissues, offering a valuable window into the early biochemical changes in IVDs (Ellingson et al., 2013, 2014; Hoppe et al., 2012). In our study, the lower T2∗ values observed in the L5/S1 segment of elite golfers suggest a possible decline in IVD health. Compared to the control group, golfers displayed lower T2∗ values in both the L5/S1 segment (49.74 ± 4.36 vs 54.08 ± 4.36 ms; *P* = 0.49) and the posterior nucleus pulposus (58.73 ± 7.43 vs 63.4 ± 7.77 ms; *P* = 0.66). This decrease in T2∗ values, although not statistically significant, aligns with a pattern of early degeneration that could precede visible structural deterioration on standard MRI scans. In particular, T2∗ values may reveal early degeneration sooner than the widely used Pfirrmann grading system, which focuses on more advanced degenerative signs. The use of T2∗ mapping could thus provide a method for early diagnosis, potentially allowing athletes to adjust their training and swing mechanics before more serious structural changes occur. This early detection capability could be essential in sports like golf, where repetitive, high-stress actions such as the golf swing place substantial mechanical loads on the lumbar spine.

The biomechanical demands of a golf swing create distinct challenges for the lumbar spine. Specifically, the swing involves considerable lateral flexion, torsion, and compressive forces, particularly during the downswing, impact, and follow-through phases (Hosea and Gatt, 1996b; Hosea et al., 1990). These phases generate peak lateral flexion and shear forces that are close to the lumbar spine's structural tolerance limits. Elite golfers, who achieve higher clubhead speeds, are especially at risk, as the resulting forces are more intense than those experienced by recreational golfers. In our study, the lower T2∗ values in elite golfers' lower lumbar segments correspond with these high-stress phases of the swing. Previous research shows that 41% of lower back injuries in golfers occur during these phases, especially during impact and early follow-through, when high compression loads are exerted on the spine (McHardy et al., 2007b; Cole and Grimshaw, 2014; McCarroll, 2001). The specific lower T2∗ values in the posterior nucleus pulposus of elite golfers further support the idea that these high forces could lead to early disc degeneration, particularly in this high-impact region of the spine. This relationship between swing mechanics and lumbar health points to the importance of understanding how subtle adjustments in swing technique could mitigate these effects. Elite golfers may benefit from swing adjustments that distribute forces more evenly across the spine or reduce lateral flexion and torsional loads. Future studies could explore whether specific swing modifications could help protect the lumbar spine without compromising performance.

The occurrence of lower back pain (LBP) among elite golfers is well-documented, and our study's ODI results suggest that elite golfers experience higher levels of LBP than the control group. This increase in pain and disability is consistent with existing literature linking golf-specific lumbar strain to chronic back issues (Lindsay and Vandervoort, 2014; Grimshaw et al., 2002; McHardy et al., 2007b). Studies have shown that golfers with LBP often exhibit different swing mechanics, including greater lateral flexion toward the lead side and reduced trunk rotation toward the trail side (Lindsay and Horton, 2002). Although these differences may not pose a direct risk during the backswing, they can disrupt proper positioning for the downswing, leading to greater strain on the lumbar spine as golfers attempt to return to the correct position. Elite golfers who suffer from LBP also tend to rotate their trunks through a range of motion that exceeds their physical limits, creating even more strain on the lumbar discs. As our study suggests, the lower T2∗ values and higher ODI scores may be linked, indicating a relationship between lumbar degeneration and increased functional impairment. This correlates well with studies demonstrating that poor spinal mobility is associated with higher lumbar spinal stresses, which are twice as high as normal conditions (Dolan and Adams, 1993). Addressing LBP in golfers may, therefore, require a holistic approach that includes assessments of spinal health and biomechanical function, as well as interventions aimed at reducing repetitive lumbar strain.

Our findings highlight the potential of T2∗ mapping as a diagnostic tool for detecting early disc changes, which could help guide injury prevention strategies. Given the high prevalence of LBP and lumbar degeneration in golfers, targeted interventions that address spinal mobility, core stability, and swing mechanics may help alleviate stress on the lower spine and prevent long-term damage. For instance, core-strengthening exercises that support the lumbar spine and enhance trunk stability could be beneficial. Specific mobility training, such as thoracic spine and hip flexibility exercises, may allow golfers to reduce excessive lumbar rotation and lateral flexion, both of which contribute to lumbar strain. Additionally, swing technique adjustments that avoid extreme lateral flexion could distribute forces more evenly and reduce torsional and compressive loads on the lumbar spine.

Golf-specific warm-up routines that prepare the lower back for high-impact forces, as well as post-swing recovery exercises, could also play a role in reducing cumulative lumbar damage over time. Given the trends identified in this study, coaches and trainers should consider incorporating these injury prevention strategies into their training regimens to safeguard golfers’ spinal health while maintaining performance.

To our knowledge, this is among the first studies to apply quantitative T2∗ relaxometry, rather than conventional morphological grading alone, to the lumbar spine of high-level amateur golfers, and the first to combine this imaging approach with a formal Monte Carlo-based simulation to estimate sample sizes required for adequately powered future trials. This dual approach represents a methodological innovation in sports-imaging research: rather than simply reporting a (likely underpowered) pilot comparison and calling for l*arger studies* in general terms, our simulation framework provides region-specific, statistically grounded sample size estimates (e.g., 20-30 participants per group for the anterior nucleus pulposus and L2-3 versus 140-180 for L5-S1 under 80% power), allowing future investigators to design studies efficiently rather than arbitrarily. This region-specific power estimation is, to our knowledge, novel in the golf injury imaging literature and could serve as a template for pilot-to-definitive-study transitions in other sport-specific musculoskeletal imaging research.

Our findings of altered T2∗ signal patterns, particularly in the lower lumbar segments, parallel observations in other athletic populations subjected to repetitive rotational or asymmetric spinal loading, such as elite rowers, who demonstrated characteristic patterns of intervertebral disc alteration on T2∗ mapping despite being asymptomatic (Benedikter et al., 2022). The convergence of findings across sports with differing biomechanical demands (rotational loading in golf vs. predominantly flexion-extension loading in rowing) but a shared outcome of early, asymptomatic disc-level change on quantitative MRI suggests that T2∗ mapping may be capturing a generalizable early signature of overuse-related disc adaptation or degeneration that precedes symptom onset across multiple sporting disciplines. This raises the broader question of whether an *athlete's disc* phenotype exists, analogous to the well-described *athlete's heart*, and whether such changes represent maladaptive degeneration, a benign adaptive remodeling response, or some combination depending on the magnitude and chronicity of loading. Longitudinal studies tracking these changes over a competitive career, ideally beginning in adolescence or early adulthood before high training loads are established, would be valuable in distinguishing these possibilities.

From a clinical standpoint, our findings raise the possibility that T2∗ mapping could eventually serve as a screening tool integrated into periodic health evaluations for elite and high-level amateur golfers, analogous to cardiac screening protocols already common in professional sport. Identification of golfers with early, asymptomatic T2∗ changes at L4/5 or L5/S1 could prompt targeted preventive interventions, such as individualized strength and conditioning programs, swing modification under biomechanical analysis, or modified training volume, before the development of clinical low back pain or structural progression to disc herniation. However, the cost, scan time, and limited availability of T2∗ mapping protocols outside specialized research centers currently limit widespread clinical adoption, and further work is needed to determine the predictive value of baseline T2∗ findings for future symptomatic disease before such screening could be justified on a cost-effectiveness basis.

### 4.1 Limitations

This study has several limitations. The primary limitation is the small sample size, which restricted the statistical power of the study and limited our ability to generalize the results. Due to strict inclusion criteria, which ensured participants were elite golfers, recruitment of a large cohort was challenging. Consequently, several differences observed between golfers and controls, particularly in the lower lumbar spine, did not reach statistical significance despite showing potentially clinically relevant trends.

To better understand the sample size requirements for future investigations, a simulation-based power analysis was performed using the observed T2∗ values. The analysis demonstrated substantial variation in the sample sizes required to detect group differences across spinal regions. The anterior nucleus pulposus and L2-3 regions showed the most favorable power characteristics, requiring only 20-30 participants per group to achieve 80% power and 30 participants per group to achieve 90% power. In contrast, the lower lumbar segments, which are biomechanically most relevant to the golf swing, required substantially larger cohorts. For the L5-S1 segment, 140-180 participants per group would be required to achieve 80% power and 200-220 for 90% power. The L3-4 segment would require 540-770 participants per group for 80% power, with the normal distributional assumption failing to reach 90% power within the simulated range. L4-5 did not reach either threshold within the simulated range of up to 1000 participants per group under any distributional assumption. Similarly, the anterior annulus fibrosus (390-420 for 80% power), posterior nucleus pulposus (370-430 for 80% power), and posterior annulus fibrosus (200-230 for 80% power) all require substantially larger cohorts than are practically achievable in a single-center study. These findings are particularly important because the L4/L5 and L5/S1 levels are widely recognized as the lumbar segments exposed to the greatest rotational, compressive, and shear forces during the golf swing. Although our pilot study identified trends toward lower T2∗ values at these levels in elite golfers, the power analysis indicates that confirmation of these findings will require large-scale, multicenter studies with substantially larger cohorts. Such studies would be better positioned to determine whether the observed reductions in T2∗ values reflect early golf-related disc degeneration and to establish the clinical relevance of these changes.

Additional limitations are related to the T2∗ mapping process itself. T2∗ mapping requires a high level of precision in ROI selection, and in this study two experienced observers manually placed the ROIs. Minor variations in slice thickness and alignment, as well as possible inclusion of non-IVD tissues, could introduce variability. Motion artifacts from the pulsating aorta and respiratory movement may also have affected image quality, although every effort was made to minimize these factors through standardized imaging conditions. Furthermore, the “magic angle effect,” where T2∗ relaxation times increase when collagen fibers align at specific angles relative to the magnetic field, may have influenced some measurements. Future studies incorporating larger cohorts, automated ROI placement, and longitudinal follow-up designs may further clarify the relationship between golf-specific biomechanical loading and early intervertebral disc degeneration.

An additional consideration not fully explored in this pilot study is the potential role of training volume and periodization in mediating disc health. Our golfer cohort reported a minimum training commitment of 10 h per week; however, we did not stratify participants by total cumulative training hours over their competitive career, nor by the specific distribution of practice (e.g., proportion of time spent on full-swing repetition with a driver versus short-game practice, which imposes substantially lower lumbar loads). Future studies should consider collecting detailed training-load histories, including total swing counts per week and per year, as a continuous variable that may correlate with T2∗ values in a dose-response manner, which would strengthen the causal inference linking golf-specific loading to disc-level change.

## 5 Conclusion

In conclusion, this pilot study provides preliminary evidence that high-level amateur golfers exhibit patterns of lumbar intervertebral disc alteration on quantitative T2∗ mapping that differ from those of matched, asymptomatic non-athletic controls, particularly in the lower lumbar segments (L4/5 and L5/S1) that bear the greatest biomechanical load during the golf swing. While the overall between-group difference in T2∗ values did not reach statistical significance, golfers demonstrated a higher frequency of elevated Pfirrmann grades, including grade 4 discs not observed in any control participant, and showed a consistent trend toward lower T2∗ values in the posterior nucleus pulposus and at L5/S1, both anatomically and biomechanically plausible sites of early degeneration given the compressive, shear, and rotational forces generated during the downswing and impact phases of the golf swing.

Several conclusions can be drawn from this work. First, the observed morphological and quantitative trends, despite the small sample size, are biomechanically coherent and anatomically localized, strengthening the plausibility that they reflect a true, golf-related phenomenon rather than random variation. Second, our Monte Carlo simulation-based power analysis demonstrates that the sample sizes required to confirm these findings vary substantially by spinal region, ranging from as few as 20–30 participants per group for the anterior nucleus pulposus and the L2-3 segment to 140–180 participants per group for the clinically most relevant L5-S1 segment, and exceeding 1000 participants per group for L4-5 under all distributional assumptions; this region-specific information should directly inform the design of future, adequately powered, multicenter studies. Third, the elevated Oswestry Disability Index scores in golfers compared with controls, although modest in absolute terms, suggest that even subclinical disc changes may be accompanied by measurable, if minor, functional impact, supporting the value of combining patient-reported outcomes with quantitative imaging in future protocols.

Looking forward, we propose that future research should pursue: (1) larger, multicenter cohorts powered according to the region-specific estimates provided here, particularly targeting the L4/5 and L5/S1 segments; (2) longitudinal designs that track T2∗ values and Pfirrmann grades over multiple seasons to determine whether early changes progress, stabilize, or regress with modified training; (3) integration of three-dimensional swing biomechanics data (including X-Factor, Crunch Factor, and ground reaction forces) with T2∗ values to directly test dose-response relationships between swing mechanics and disc-level change; (4) inclusion of female golfers and a wider age range, as our cohort was limited to male golfers aged 22–42; and (5) exploration of T2∗ mapping as a practical screening tool within existing athlete health monitoring programs, alongside cost-effectiveness analyses.

Ultimately, by combining quantitative biochemical MRI with rigorous statistical planning for future research, this study contributes both substantive preliminary findings and a methodological framework that may accelerate the development of evidence-based guidelines for preserving lumbar spine health in golfers, a population in which early detection and targeted intervention could meaningfully extend competitive careers and improve long-term musculoskeletal health.

## Author contributions statement

Credit for authorship should be based on: [1] substantial contributions to research design, or the acquisition, analysis or interpretation of data; [2] approval of the submitted and final versions.

## Ethics approval

This study was approved by the local ethics committee (Register number 2021-1512) and was conducted according to the revised declaration of Helsinki.

Consent to participate and for publication was conducted in written form by all participants.

## Funding

Funding was conducted by Deutsche Arthrosehilfe e.V. (19.000,00 Euro).

## Conflicts of interest

MP none.

BB none.

TM none.

DA none.

## Glossary

Oswestry Disability Index: (ODI)
Lower Back Pain: (LBP)
Magnetic Resonance Imaging: (MRI)
Intervertebral Disk: (IVD)
Region of Interest: (ROI)

## References

[bib1] Arnold B.F., Hogan D.R., Colford J.M., Hubbard A.E. (2011). Simulation methods to estimate design power: an overview for applied research. BMC Med. Res. Methodol..

[bib2] Baranto A., Hellström M., Cederlund C.G., Nyman R., Swärd L. (2009). Back pain and MRI changes in the thoraco-lumbar spine of top athletes in four different sports: a 15-year follow-up study. Knee Surg. Sports Traumatol. Arthrosc..

[bib3] Benedikter C., Abrar D.B., Konieczny M., Schleich C., Bittersohl B. (2022). Patterns of intervertebral disk alteration in asymptomatic elite rowers: a T2∗ MRI mapping study. Orthop. J. Sports Med..

[bib6] Bittersohl B., Benedikter C., Franz A. (2019). Elite rowers demonstrate consistent patterns of hip cartilage damage compared with matched controls: a T2∗ mapping study. Clin. Orthop. Relat. Res..

[bib5] Bittersohl B., Miese F.R., Dekkers C. (2013). T2∗ mapping and delayed gadolinium-enhanced magnetic resonance imaging in cartilage (dGEMRIC) of glenohumeral cartilage in asymptomatic volunteers at 3 T. Eur. Radiol..

[bib4] Bittersohl B., Miese F.R., Hosalkar H.S. (2012). T2∗ mapping of hip joint cartilage in various histological grades of degeneration. Osteoarthr. Cartil..

[bib9] Bourgain M., Rouch P., Rouillon O., Thoreux P., Sauret C. (2022). Golf swing biomechanics: a systematic review and methodological recommendations for kinematics. Sports.

[bib10] Burdorf A., Van Der Steenhoven G.A., Tromp-Klaren E.G. (1996). A one-year prospective study on back pain among novice golfers. Am. J. Sports Med..

[bib11] Chu Y., Sell T.C., Lephart S.M. (2010). The relationship between biomechanical variables and driving performance during the golf swing. J. Sports Sci..

[bib12] Cole M.H., Grimshaw P.N. (2014). The crunch factor's role in golf-related low back pain. Spine J..

[bib13] Dolan P., Adams M.A. (1993). Influence of lumbar and hip mobility on the bending stresses acting on the lumbar spine. Clin. Biomech..

[bib14] Ellingson A.M., Mehta H., Polly D.W., Ellermann J., Nuckley D.J. (2013). Disc degeneration assessed by quantitative T2∗ (T2 star) correlated with functional lumbar mechanics. Spine.

[bib15] Ellingson A.M., Nagel T.M., Polly D.W., Ellermann J., Nuckley D.J. (2014). Quantitative T2∗ (T2 star) relaxation times predict site specific proteoglycan content and residual mechanics of the intervertebral disc throughout degeneration. J. Orthop. Res..

[bib16] Fairbank J.C., Couper J., Davies J.B., O'Brien J.P. (1980). The Oswestry low back pain disability questionnaire. Physiotherapy.

[bib17] Farrally M.R., Cochran A.J., Crews D.J., Hurdzan M.J., Price R.J., Snow J.T., Thomas P.R. (2003). Golf science research at the beginning of the twenty-first century. J. Sports Sci..

[bib18] Gluck G.S., Bendo J.A., Spivak J.M. (2008). The lumbar spine and low back pain in golf: a literature review of swing biomechanics and injury prevention. Spine J..

[bib19] Gosheger G., Liem D., Ludwig K., Greshake O., Winkelmann W. (2003). Injuries and overuse syndromes in golf. Am. J. Sports Med..

[bib20] Grimshaw P.N., Giles A., Tong R. (2002). Low back and elbow injuries in golf. Sports Med..

[bib21] Haacke E.M., Mittal S., Wu Z., Neelavalli J., Cheng Y.C. (2009). Susceptibility-weighted imaging: technical aspects and clinical applications, part 1. AJNR Am J Neuroradiol.

[bib23] Hesper T., Hosalkar H.S., Bittersohl D., Welsch G.H., Krauspe R., Zilkens C., Bittersohl B. (2014). T2∗ mapping for articular cartilage assessment: principles, current applications, and future prospects. Skelet. Radiol..

[bib24] Hoppe S., Quirbach S., Mamisch T.C., Krause F.G., Werlen S., Benneker L.M. (2012). Axial T2∗ mapping in intervertebral discs: a new technique for assessment of intervertebral disc degeneration. Eur. Radiol..

[bib27] Hosea T.M., Gatt C.J., Galli K.M., Cochran A.J. (1990). Science and Golf: Proceedings of the First World Scientific Congress of Golf.

[bib25] Hosea T.M., Gatt C.J. (1996). Back pain in golf. Clin. Sports Med..

[bib26] Hosea T.M., Gatt C.J. (1996). Back pain in golf. Clin. Sports Med..

[bib29] Kolf A.K., Konieczny M., Hesper T. (2019). T2∗ mapping of the adult intervertebral lumbar disc: normative data and analysis of diurnal effects. J. Orthop. Res..

[bib30] Lindsay D.M., Horton J.F. (2002). Comparison of spine motion in elite golfers with and without low back pain. J. Sports Sci..

[bib31] Lindsay D.M., Vandervoort A.A. (2014). Golf-related low back pain: a review of causative factors and prevention strategies. Asian J. Sports Med..

[bib32] McCarroll J.R. (2001). Overuse injuries of the upper extremity in golf. Clin. Sports Med..

[bib33] McHardy A., Pollard H., Luo K. (2006). Golf injuries: a review of the literature. Sports Med..

[bib34] McHardy A., Pollard H., Luo K. (2007). One-year follow-up study on golf injuries in Australian amateur golfers. Am. J. Sports Med..

[bib35] McHardy A., Pollard H., Luo K. (2007). Golf-related lower back injuries: an epidemiological survey. J. Chiropract. Med..

[bib36] Mosher T.J., Dardzinski B.J., Smith M.B. (2000). Human articular cartilage: influence of aging and early symptomatic degeneration on the spatial variation of T2 — preliminary findings at 3 T. Radiology.

[bib38] Murray A., Junge A., Robinson P.G., Bizzini M., Sutton L., MacInnis P., Forde A., Roberts C., Phillips G. (2020). International consensus statement on injury and illness surveillance methodology in golf, including a golf-specific definition of injury and illness. Br. J. Sports Med..

[bib39] Murray A., Junge A., Robinson P.G., Clarsen B., Mountjoy M.L., Drobny T., Gill L., Gazzano F., Voight M., Dvorak J. (2023). Cross-sectional study of characteristics and prevalence of musculoskeletal complaints in 1170 male golfers. BMJ Open Sport Exerc Med.

[bib37] Murray A.D., Daines L., Archibald D., Hawkes R.A., Schiphorst C., Kelly P., Grant L., Mutrie N. (2017). The relationships between golf and health: a scoping review. Br. J. Sports Med..

[bib40] Pfirrmann C.W., Metzdorf A., Zanetti M., Hodler J., Boos N. (2001). Magnetic resonance classification of lumbar intervertebral disc degeneration. Spine.

[bib41] PGA Tour Driving distance statistics. https://www.pgatour.com/stats/detail/02401.

[bib42] Roberts S., Evans H., Trivedi J., Menage J. (2006). Histology and pathology of the human intervertebral disc. J Bone Joint Surg Am.

[bib43] Robinson P.G., Clarsen B., Murray A., Junge A., Mountjoy M.L., Drobny T., Gill L., Gazzano F., Voight M., Dvorak J. (2024). A prospective study of injuries and illnesses among 910 amateur golfers during one season. BMJ Open Sport Exerc Med.

[bib44] Sugaya H., Morgan D.A., Banks S.A. (1996). Sun Valley, ID.

[bib45] Sward L., Hellstrom M., Jacobsson B.O., Nyman R.M., Peterson L. (1991). Disc degeneration and associated abnormalities of the spine in elite gymnasts: a magnetic resonance imaging study. Spine.

[bib46] Witwit W.A., Kovac P., Sward A. (2018). Disc degeneration on MRI is more prevalent in young elite skiers compared to controls. Knee Surg. Sports Traumatol. Arthrosc..

[bib48] Zouzias I.C., Hendra J., Stodelle J., Limpisvasti O. (2018). Golf injuries: epidemiology, pathophysiology, and treatment. J. Am. Acad. Orthop. Surg..

